# Ser7 Phosphorylation of the CTD Recruits the RPAP2 Ser5 Phosphatase to snRNA Genes

**DOI:** 10.1016/j.molcel.2011.11.006

**Published:** 2012-01-13

**Authors:** Sylvain Egloff, Justyna Zaborowska, Clélia Laitem, Tamás Kiss, Shona Murphy

**Affiliations:** 1 Sir William Dunn School of Pathology, University of Oxford, Oxford OX1 3RE, UK; 2 Université de Toulouse, UPS, Laboratoire de Biologie Moléculaire Eucaryote, F-31000 Toulouse, France; 3 CNRS, LBME, F-31000 Toulouse, France

## Abstract

The carboxy-terminal domain (CTD) of the large subunit of RNA polymerase II (Pol II) comprises multiple heptapeptide repeats of the consensus Tyr1-Ser2-Pro3-Thr4-Ser5-Pro6-Ser7. Reversible phosphorylation of Ser2, Ser5, and Ser7 during the transcription cycle mediates the sequential recruitment of transcription/RNA processing factors. Phosphorylation of Ser7 is required for recruitment of the gene type-specific Integrator complex to the Pol II-transcribed small nuclear (sn)RNA genes. Here, we show that RNA Pol II-associated protein 2 (RPAP2) specifically recognizes the phospho-Ser7 mark on the Pol II CTD and also interacts with Integrator subunits. siRNA-mediated knockdown of RPAP2 and mutation of Ser7 to alanine cause similar defects in snRNA gene expression. In addition, we show that RPAP2 is a CTD Ser5 phosphatase. Taken together, our results indicate that during transcription of snRNA genes, Ser7 phosphorylation facilitates recruitment of RPAP2, which in turn both recruits Integrator and dephosphorylates Ser5.

## Highlights

► RPAP2 interacts both with Pol II and the snRNA gene-specific Integrator complex ► RPAP2 recognizes the Ser7P mark on the Pol II CTD ► RPAP2 is required for expression of snRNA and protein-coding genes ► RPAP2 is a Ser5P-specific CTD phosphatase

## Introduction

In human cells, transcription of protein-coding genes and most small nuclear (sn)RNA genes is carried out by RNA polymerase (Pol) II. The largest subunit of Pol II, Rpb1, contains an unusual carboxy-terminal domain (CTD) consisting of tandem repeats of the consensus sequence Tyr1-Ser2-Pro3-Thr4-Ser5-Pro6-Ser7. The CTD is an evolutionarily conserved structure that can be extensively and reversibly phosphorylated in vivo (Chapman et al., 2008; Egloff and Murphy, 2008). CTD phosphorylation has been implicated in key steps of transcription such as preinitiation complex (PIC) assembly, promoter clearance, promoter proximal pausing, transcript elongation, and polymerase recycling (Buratowski, 2009). In addition, cotranscriptional processing of nascent RNAs is affected by the phosphorylation status of the CTD. It is well established that the CTD acts as a molecular platform allowing the transcription/RNA processing factors to be recruited to the transcribing polymerase at the right point of the transcription cycle (Buratowski, 2003; Egloff and Murphy, 2008; Perales and Bentley, 2009). The CTD of Pol II recruited to the PIC at the promoter is hypophosphorylated. Phosphorylation of the CTD on Ser5 accompanies initiation/promoter clearance by Pol II, while subsequent phosphorylation on Ser2 occurs later in the transcription cycle, during elongation (Buratowski, 2009). A complicated interplay between CTD kinases and CTD phosphatases generates the complex phosphorylation pattern along genes, which underpins the CTD code (Buratowski, 2003; Egloff and Murphy, 2008). The Cdk7 catalytic subunit of the TFIIH complex is the main Ser5 kinase, while the Cdk9 catalytic subunit of the P-TEFb complex is responsible for Ser2 phosphorylation. Dynamic dephosphorylation of Ser2 and Ser5 is thought to make a significant contribution to the changes in CTD phosphorylation patterns during the transcription cycle and is essential for recycling Pol II (Buratowski, 2009; Egloff and Murphy, 2008). The evolutionarily conserved protein Fcp1, which is essential in yeast, dephosphorylates phospho-Ser2 preferentially (Hausmann and Shuman, 2002), whereas Ssu72 and the more recently described Rtr1 in yeast, and SCP1 in mammals, specifically dephosphorylate Ser5 (Krishnamurthy et al., 2004; Mosley et al., 2009; Yeo et al., 2003).

In addition to Ser2 and Ser5, Ser7 of the CTD is phosphorylated during transcription (Chapman et al., 2007). Ser7 phosphorylation has been detected on both protein-coding genes and the Pol II-transcribed snRNA genes (Chapman et al., 2007; Egloff et al., 2007, 2009). The role of this modification on protein-coding genes remains poorly understood, since mutation of Ser7 to alanine does not significantly affect mRNA production from several tested genes (Chapman et al., 2007; Egloff et al., 2007). In contrast, Ser7 is required for efficient transcription of snRNA genes and processing of the transcripts (Egloff et al., 2007). Human snRNA genes transcribed by Pol II are structurally different from protein-coding genes (Egloff et al., 2008; Hernandez, 2001). snRNAs are neither spliced nor polyadenylated, and instead of a poly(A) signal, the genes contain a conserved 3′ box RNA processing element (Hernandez, 1985) recognized by the snRNA gene-specific Integrator RNA 3′ end processing complex (Baillat et al., 2005). Importantly, the Integrator complex binds to the Pol II CTD, providing a molecular link between transcription and 3′ end processing. Phosphorylated Ser7 (Ser7P) is a key determinant for the recruitment of the Integrator complex to snRNA genes (Baillat et al., 2005; Egloff et al., 2007). In combination with Ser2P, it creates what may be an snRNA gene-specific double mark recognized by the Integrator complex (Egloff et al., 2010). In *Saccharomyces cerevisiae*, Ser7P is found at gene promoters and persists within the encoding region (Mayer et al., 2010; Tietjen et al., 2010), and appears to be enriched over 5′ ends, introns, and 3′ ends (Kim et al., 2010). In mammals, Ser7P is highest at the promoter region of snRNA genes but enriched toward the 3′ end of protein-coding genes (Chapman et al., 2007; Egloff et al., 2009). Recent studies indicate that Cdk7 is the major Ser7 kinase in vivo, while the Ser7 phosphatase remains to be identified (Akhtar et al., 2009; Boeing et al., 2010; Glover-Cutter et al., 2009; Kim et al., 2009). In addition to Cdk7, Bur1 kinase appears to act as a Ser7 kinase on elongating Pol II in yeast (Tietjen et al., 2010), Cdk9 can phosphorylate Ser7 in vitro (Glover-Cutter et al., 2009; Kim et al., 2009), and DNA-PK has been reported to be the major Ser7 kinase in HeLa cell nuclear extract (Egloff et al., 2010).

A recent systematic analysis determined the composition and organization of the soluble Pol II machinery (Jeronimo et al., 2007). This map of interactions reveals previously unknown components of Pol II transcription complexes. Newly identified proteins occupying key positions in this network were named RNA Pol II-associated protein (RPAP) (Jeronimo et al., 2007). Of these, the RPAP2 protein was found to interact also with the Integrator complex, raising the possibility that it may participate in expression of snRNA genes. Interestingly, the yeast homolog of RPAP2 is the Rtr1 Ser5 phosphatase, which also interacts genetically with the Pol II machinery (Gibney et al., 2008). Upon deletion of Rtr1, the Ser5P form of Pol II accumulates in whole-cell extracts and throughout mRNA gene-coding regions (Mosley et al., 2009).

Prompted by these findings, we have investigated the role of the human RPAP2 protein in snRNA gene expression. We show that RPAP2 specifically associates with Ser7-phosphorylated Pol II through a direct interaction with the CTD. Functional characterization of the protein demonstrates that, like its homolog in yeast, RPAP2 acts as a Ser5 phosphatase in vitro and Ser5P levels increase in vivo when RPAP2 is knocked down. In addition, depletion of RPAP2 affects snRNA gene expression in the same way as mutation of Ser7. Interestingly, RPAP2 does not appear to be recruited to protein-coding genes through Ser7 phosphorylation. Our results suggest that Ser7P specifically leads to Integrator recruitment and Ser5 dephosphorylation of the Pol II CTD during transcription of snRNA genes through recruitment of the RPAP2 phosphatase.

## Results

### Human RPAP2 Exists in at Least Two Distinct Complexes

**Figure 1:**
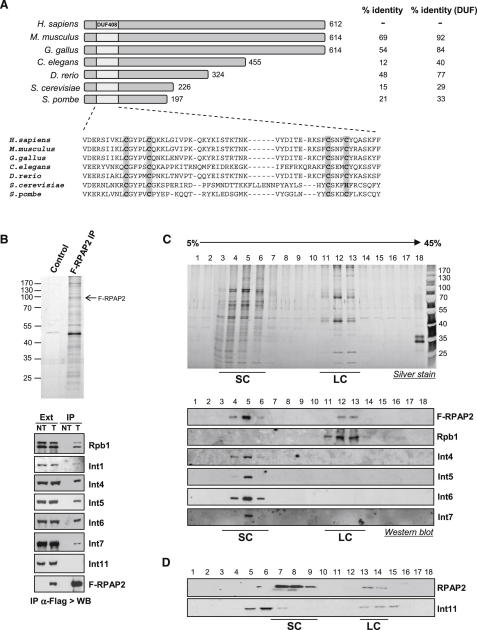
RPAP2 Exists in Two Distinct Complexes (A) Homologs of RPAP2 from the indicated species are shown on the diagram. Percent identity with RPAP2 and the DUF408 domain is indicated at the right. The DUF408 domain sequence was aligned to its mouse (*M. musculus*, NP_659160), chicken (*G. gallus*, XP_422341), *C. elegans* (CAA78048), zebrafish (*D. rerio*, AAH95735), *S. cerevisiae* (P40084), and *S. pombe* (NP_594445) homologs. Conserved cysteine residues are highlighted. (B) SDS gel showing RPAP2 affinity eluate prepared from F-RPAP2-transfected cells. Nontransfected HeLa cells were used as a control for the Flag purification. Western blots are shown confirming the presence of Rpb1, Int1, Int4, Int5, Int6, Int7, and F-RPAP2 but not Int11 in the eluate. (C) Fractionation of the F-RPAP2 eluate by ultracentrifugation on a glycerol gradient. Distribution of F-RPAP2, Rpb1, Int4, Int5, Int6, and Int7 in the gradient fractions was monitored by western blot using antibodies to the proteins noted at the right. SC, small complexes; LC, large complexes. (D) Fractionation of HeLa cell extract on glycerol gradient. Distribution of endogenous RPAP2 and Int11 was monitored by western blot. See also Figure S1.

In a recent study of the protein interaction network of the human transcription machinery, newly identified proteins were named RNA Pol II-associated protein (RPAP), based on their physical interaction with Pol II (Jeronimo et al., 2007). RPAP2 contains 612 amino acids, while the yeast homolog Rtr1 contains only 226 and 197 amino acids in *S. cerevisiae* and *S. pombe*, respectively (Figure 1A). An evolutionary conserved domain of unknown function, called DUF408, is located at the N terminus of the protein. Conservation drops significantly outside of this region, implying that this domain is required for RPAP2 function. To investigate the function of RPAP2, the protein was Flag tagged (F-RPAP2), transiently expressed in HeLa cells, and immunopurified with an anti-Flag antibody. Proteins recovered in the eluate were then visualized by silver staining and analyzed by western blotting (Figure 1B). In agreement with data from large-scale proteomics studies in human and yeast (Jeronimo et al., 2007; Mosley et al., 2009), RPAP2 strongly interacts with Rpb1, the largest subunit of Pol II. The presence of the IIa and IIo forms, corresponding to hypo- and hyperphosphorylated CTD, respectively, suggests that RPAP2 associates with transcriptionally active Pol II. A recent genome-wide study placed RPAP2 at the interface between Pol II and regulatory complexes such as Integrator (Jeronimo et al., 2007). Accordingly, the interaction between RPAP2 and the Int1, Int4, Int5, Int6, and Int7 subunits of the Integrator complex was also confirmed (Figure 1B). Integrator is a macromolecular multisubunit complex that binds to the Pol II CTD and directs 3′ end formation of snRNAs (Baillat et al., 2005; Ezzeddine et al., 2011; Tao et al., 2009). Interestingly, the catalytic subunit of the Integrator complex, Int11 (Baillat et al., 2005), does not coprecipitate with RPAP2, demonstrating that all Integrator subunits are not necessarily present in one complex. To begin deciphering the polypeptide composition of RPAP2-containing complexes, the F-RPAP2 eluate was analyzed by glycerol gradient fractionation (Figure 1C). This analysis revealed that RPAP2 and Rpb1 elute together in a large complex (LC) peaking in fractions 11–13, while a significant fraction of F-RPAP2 elutes as a smaller complex (SC), peaking at fractions 4–6, devoid of Rpb1 but containing Int4, Int5, Int6, and Int7. RPAP2 in nuclear extract also fractionates into two complexes (Figure 1D). In accordance with the absence of Int11 from the F-RPAP2 eluate, Int11 does not cofractionate with the smaller RPAP2 complex (Figure 1D). The composition of the SC and the LC was determined by mass spectrometry (See Figure S1 and Tables S1 and S2, available with this article online). While Pol II subunits were found to be major components of the LC, the SC contains a different set of proteins, including members of the chaperonin-containing TCP1 complex, XAB1 and GPN3, previously shown to interact with RPAP2 (Jeronimo et al., 2007). The presence of the Integrator complex in the SC was confirmed through identification of the DDX26/INT6 subunit, which is consistent with the previously described reciprocal interaction (Malovannaya et al., 2010). Together, our data suggest that a large fraction of RPAP2 associates with chaperone/scaffolding proteins and regulatory complexes such as Integrator. However, Pol II subunits were found to be the major proteins to interact with both Integrator complex (Malovannaya et al., 2010) and RPAP2 (Figure S1), suggesting that a complex containing Pol II, Integrator, and RPAP2 would be transient.

### The DUF408 Domain of RPAP2 Is Involved in Establishment of Protein-Protein Interactions

**Figure 2:**
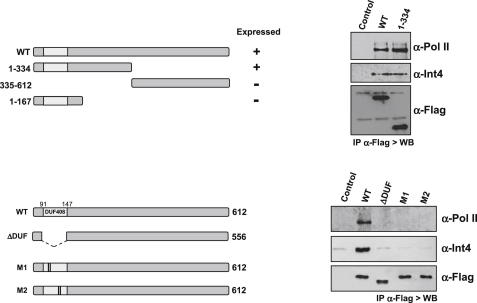
The DUF408 Domain of RPAP2 Is Involved in Establishment of Protein-Protein Interactions The series of Flag-tagged RPAP2 mutants diagrammed on the left were expressed in HeLa cells. Anti-Flag IPs were analyzed by western blot using antibodies to Pol II, Int4, and Flag (right panel). See also Figure S2.

To identify the region(s) of RPAP2 involved in Pol II and Integrator binding, truncated Flag-tagged RPAP2 proteins were expressed in HeLa cells and immunopurified with an anti-Flag antibody. Interaction with Rpb1 and the Int4 subunit of Integrator complex was then monitored by western blot (Figure 2). The N-terminal region of the protein (1–334) interacts with both Rpb1 and Int4 as efficiently as the full-length RPAP2 (WT), demonstrating that it contains all the elements required for binding. Neither the C terminus (335–612) of RPAP2 nor the further truncated N terminus (1–167) was efficiently expressed. Deletion of the 57 amino acid-long DUF408 domain completely abolishes the binding of both Pol II and Integrator. Analysis of the primary amino acid sequence of the DUF408 domain revealed a cysteine-rich motif reminiscent of a zinc finger. This motif is highly conserved with a particular conservation of four cysteine residues (Figure 1A), which are essential for function of the yeast Rtr1 protein (Gibney et al., 2008). We therefore constructed two double point mutations in the cysteine residues within the RPAP2 sequence (M1, C100A-C105A; M2, C136A-C140A). Replacement of either cysteine module completely blocks the interaction with Rpb1 and Int4, suggesting that the putative Zn finger structure is directly involved in establishment of protein-protein interactions.

Using immunofluorescence, it was determined that, under normal conditions, all F-RPAP2 proteins are mainly cytoplasmic (Figure S2). The yeast counterpart of RPAP2, Rtr1, has been shown to constitutively shuttle between the cytoplasm and the nucleus (Gibney et al., 2008). Blocking CRM1-dependent nuclear export with LMB induced a partial relocation of RPAP2, ΔDUF, M1, and M2 mutant proteins to the nucleus (Figure S2), demonstrating that these proteins all shuttle between nucleus and cytoplasm in a CRM1-dependent manner. Thus, lack of interaction with Pol II and Integrator complex cannot be explained by mislocalization of the mutant proteins.

### RPAP2 Interacts with Transcribing Pol II Phosphorylated on Ser7

**Figure 3:**
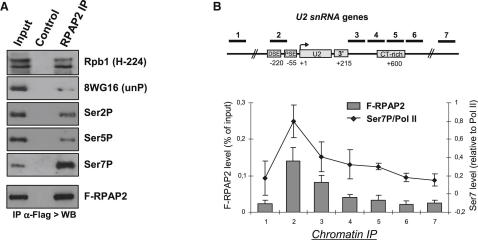
RPAP2 Interacts with Ser7-Phosphorylated Pol II (A) Western blot analysis of F-RPAP2 eluate using antibodies directed against the different phosphorylated forms of Pol II. The amount of total Rpb1 (H-224) was equalized to the input. Extract from nontransfected cells was used as a control. (B) ChIP assay was performed using anti-Flag antibody against F-RPAP2 (gray bars). The Ser7P/Pol II profile (Egloff et al., 2009) is shown as a line. The regions amplified are noted on the diagram of U2 snRNA genes above. Error bars indicate the standard deviation obtained from at least three independent experiments. See also Figure S3.

RPAP2 is associated with the IIa and IIo forms of Pol II, corresponding to hypophosphorylated and hyperphosphorylated CTD, respectively. In an attempt to further characterize this interaction, the CTD phosphorylation status of RPAP2-associated Pol II was investigated by western blot using antibodies specific to unphosphorylated CTD (8WG16), phospho-Ser2, phospho-Ser5, and phospho-Ser7 (Figure 3A). After equalization of the amount of copurified Pol II in the input, RPAP2 was found to weakly interact with unphosphorylated Pol II, whereas Ser2P and Ser5P are readily detected in the IP, suggesting that RPAP2 interacts with transcriptionally engaged Pol II. Unexpectedly, we found that the major CTD modification of RPAP2-associated Pol II is the Ser7P mark. The function of this CTD modification in expression of mammalian genes remains poorly understood, since so far only a role in expression of snRNA genes has been described (Egloff et al., 2007).

To test whether RPAP2 is recruited to snRNA genes during the transcription cycle, we analyzed its association with the *U2 snRNA* genes by chromatin immunoprecipitation (ChIP) using antibodies to either the Flag epitope at the N terminus (Figure 3B) or three tandem Myc epitopes at the C terminus (Figure S3). Transcription from *U2 snRNA* genes has been extensively characterized (Egloff et al., 2009). These genes have specialized TATA-less promoters and a snRNA gene-specific RNA 3′ processing element, the 3′ box, located immediately downstream of the snRNA-encoding region (Figure 3B, see diagram). Although transcription extends for up to 1 kb downstream of the start site (Cuello et al., 1999), ChIP analysis indicates that RPAP2 is preferentially associated with the promoter (Figure 3B) and RNA-encoding region (Figure S3) of *U2 snRNA* genes. Interestingly, we previously showed that Pol II transcribing *U2 snRNA* genes is most highly phosphorylated on Ser7 of the CTD early in transcription (Egloff et al., 2009). These results support the notion that RPAP2 preferentially associates with Ser7-phosphorylated Pol II and further suggests that Ser7P may be a critical determinant for its association with the polymerase machinery.

### Ser7 of the Pol II CTD Is Required for Interaction with RPAP2 In Vivo and In Vitro

**Figure 4:**
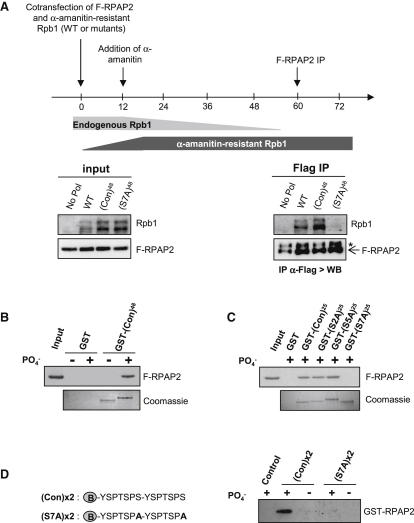
Ser7 of the Pol II CTD Is Required for Interaction with RPAP2 In Vivo and In Vitro (A) F-RPAP2 IP after ectopic expression of α-amanitin-resistant Rpb1. The timing of the experiment is noted in the diagram above. Detection of Rpb1 and F-RPAP2 in the input (left) and the IP (right) by western blot analysis is shown in the bottom panel. (Con) designates consensus CTD heptapeptides. (B) F-RPAP2 eluate was incubated with GST alone or GST-CTD-(Con)^48^, phosphorylated or not, and RPAP2 binding was monitored by western blot analysis. The bottom panel represents Coomassie blue staining of the input GST-CTD. (C) F-RPAP2 eluate was incubated with in vitro-phosphorylated GST-CTD and RPAP2 binding to the WT CTD (Con)^25^ and mutant (Ser2A/Ser5A/Ser7A)^25^ CTDs assayed by western blot analysis. The bottom panel represents Coomassie blue staining of the input GST-CTD. (D) The structures of the biotinylated peptides used are shown on the left. Binding of recombinant GST-RPAP2 to the peptide noted above the lane, phosphorylated or not, was monitored by western blot. For the control, beads were incubated with RPAP2 in the absence of peptide. See also Figure S4.

To determine whether RPAP2 requires Ser7 of the CTD to associate with Pol II in vivo, we used the CTD complementation system in which mutations are introduced into consensus (Con) CTD repeats in an α-amanitin-resistant Pol II large subunit (Rpb1) (Chapman et al., 2005; Egloff et al., 2007; Gerber et al., 1995). A CTD with 48 consensus repeats ([Con]^48^) was used for complementation because this properly supports viability (Chapman et al., 2005, 2007). The large subunit of endogenous Pol II is turned over within 48 hr of α-amanitin addition, as confirmed by western blot analysis (Figure 4A, left panel, no Pol). α-amanitin-resistant Rpb1 containing the WT, [(Con)^48^] and [(S7A)^48^] CTD are easily detected after α-amanitin treatment (Figure 4A, left panel). Forty-eight hours after addition of α-amanitin, RPAP2 was immunoprecipitated, and its interaction with α-amanitin-resistant Pol II was tested by western blot. While the WT and the (Con)^48^ Pol II are efficiently pulled down, the (S7A)^48^ Pol II fails to efficiently interact with RPAP2 (Figure 4A, right panel). Importantly, the same amount of F-RPAP2 was recovered after IP (bottom panel). Thus, Pol II lacking Ser7 of the CTD is unable to efficiently associate with RPAP2 in vivo, demonstrating that Ser7 is required for this interaction.

To determine whether RPAP2 can associate with the CTD in vitro, we performed glutathione S-transferase (GST) pull-down analysis with GST-CTD carrying 48 consensus heptapeptide repeats and the F-RPAP2 eluate from Figure 1B. Western blot analysis of the pull-down demonstrates that RPAP2 binds strongly to the CTD only after phosphorylation (Figure 4B). Phosphorylation on Ser2, Ser5, and Ser7 is detected after in vitro phosphorylation of the CTD (Egloff et al., 2007). Mutation of Ser7 to alanine has a drastic effect on RPAP2 binding, demonstrating that this residue is critical for the interaction (Figure 4C). In contrast, mutation of Ser2 or Ser5 does not affect significantly the binding of RPAP2. These results strongly suggest that phosphorylation of Ser7 is the key determinant for RPAP2 binding. Interestingly, Int4 shows a similar specific requirement for CTD phosphorylation and Ser7 (Figure S4), supporting the notion that RPAP2 mediates the interaction between the CTD and this Integrator subunit.

N-terminally biotinylated CTD peptides consisting of only two heptapeptide repeats were also tested for their ability to interact with bacterially expressed GST-RPAP2 before and after in vitro phosphorylation (Figure 4D). In accordance with the previous results, RPAP2 does not interact with nonphosphorylated peptides. In contrast, recombinant RPAP2 efficiently interacts with an in vitro phosphorylated CTD peptide comprising two consensus repeats [(Con)×2], demonstrating that the interaction between RPAP2 and the CTD is direct and that two CTD repeats are sufficient for binding. Importantly, an in vitro phosphorylated CTD carrying two repeats with alanine instead of serine at positions 7 [(S7A)×2] does not interact with RPAP2. Together, these results indicate that RPAP2 directly interacts with the phospho-CTD through Ser7.

### RPAP2 Is Required for Proper Expression of Pol II-Transcribed snRNA Genes

**Figure 5:**
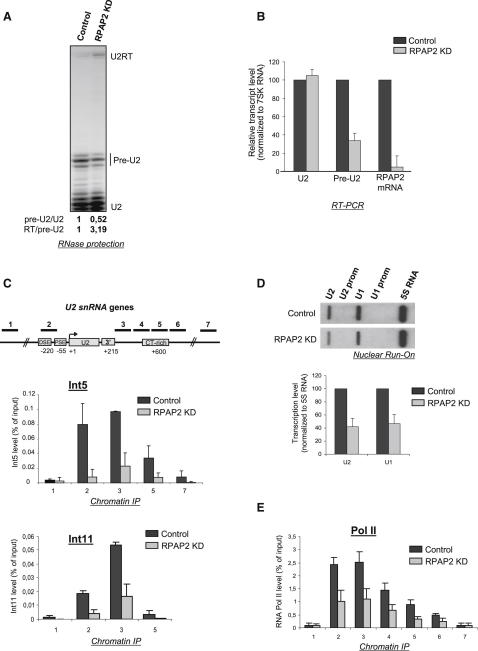
RPAP2 Is Required for Proper Expression of Pol II-Transcribed snRNA Genes (A) RNase protection analysis of transcripts from endogenous *U2 snRNA* genes in control cells or cells transfected with an siRNA specific for RPAP2 (left panel). The different RNA species are noted. (B) qRT-PCR analysis of U2, pre-U2, and RPAP2 mRNA in total RNA normalized to the Pol III-transcribed 7SK RNA. (C) qPCR quantitation of ChIP analysis of *U2 snRNA* genes using antibodies against the Int5 and Int11 subunits of the Integrator complex, after RPAP2 knockdown. The regions amplified are noted on the diagram above. (D) Nuclear run-on analysis with and without RNAi-mediated knockdown (KD) of RPAP2 is shown. An oligonucleotide complementary to transcripts from the Pol III-transcribed *5S RNA* gene was used as a control for the level of transcription. Corrected hybridization signals are shown in the graph below. (E) qPCR quantitation of ChIP analysis of *U2 snRNA* genes using an antibody against Pol II occupancy, with or without RPAP2 knockdown. Regions amplified are as shown in (C). Error bars indicate the standard deviation obtained from at least three independent experiments. See also Figure S5.

**Figure 6:**
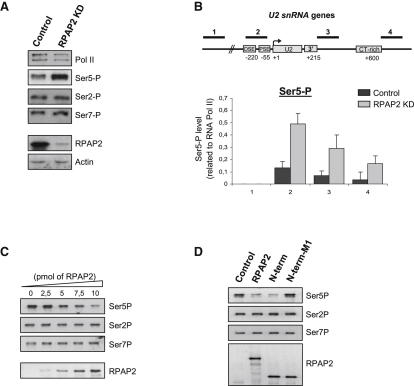
RPAP2 Is a CTD Phosphatase (A) Western blot analysis of whole-cell extracts from control cells or cells transfected with an siRNA specific for RPAP2. Antibodies used are noted on the right. (B) qPCR quantitation of ChIP analysis of *U2 snRNA* genes after RPAP2 knockdown using an antibody against Ser5P, relative to Pol II signal. Primers used are noted on the diagram of *U2 snRNA* genes above. Error bars indicate the standard deviation obtained from at least three independent experiments. (C) Western blot analysis of RPAP2 phophatase assay performed using P-TEFb-phosphorylated GST-CTD as a substrate. Increasing amounts of recombinant RPAP2 (bottom panel) were incubated with phosphorylated CTD, and phosphatase activity was visualized using antibodies directed against Ser5P, Ser2P, and Ser7P. (D) Western blot analysis of RPAP2 phophatase assay performed using mutant RPAP2 proteins. Phosphatase activity of full-length, N-terminal region (N-term), and the mutant N-terminal region (N-term-M1) RPAP2 proteins, using phosphorylated CTD as substrate, was visualized using antibodies directed against Ser5P, Ser2P, and Ser7P. See also Figure S6.

We previously showed that Ser7P is critical for transcription and 3′ end processing of snRNA genes (Egloff et al., 2007). Since Ser7P is required for RPAP2 recruitment to the Pol II machinery and RPAP2 is recruited to snRNA genes, we asked whether RPAP2 participates in snRNA gene expression. The level of RPAP2 was knocked down by RNAi and the effect on expression of the *U2 snRNA* analyzed by RNase protection (Figure 5A). The efficiency of the knockdown was verified by measuring the level of both RPAP2 mRNA (Figure 5B) and the protein (see Figure 6A). Two main RNase protection products, corresponding to the U2 pre-snRNA and the stable mature *U2 snRNA* (Egloff et al., 2007), are detected. A third minor RNA species corresponds to the readthrough product (U2RT) that has escaped 3′ box-directed processing (Medlin et al., 2005). The ratio of short-lived pre-U2 to the very stable mature U2 (U2) (Fury and Zieve, 1996), is reduced to approximately 50% when RPAP2 is knocked down (Figure 5A). qRT-PCR analysis of the RNA also indicates that the level of pre-U2 decreases to approximately 40% when RPAP2 is knocked down (Figure 5B). In addition, the ratio of RT to pre-U2 products increases more than 3-fold when RPAP2 is knocked down (Figure 5A), indicating that recognition of the 3′ box is affected, as would be expected if Integrator recruitment is impaired. The Int5 subunit of the Integrator complex is readily detectable across the whole transcribed region of U2 snRNA genes and is enriched at the promoter and 3′ box (Figure 5C). RPAP2 knockdown results in a dramatic reduction of the level of Int5 (Figure 5C), with a drop of up to 80% when related to the level of Pol II (Figure S5A). Interestingly, RPAP2 knockdown also affects the recruitment of the catalytic Int11 subunit (Figure 5C and Figure S5A), suggesting that recruitment of other Integrator subunits, together with RPAP2, is a prerequisite for Int11 association to snRNA genes. In support of this, Int4 knockdown (Figure S5B) affects 3′ processing and reduces Int11 association (Figures S5C and S5E). We conclude that RPAP2 plays an important role in recruiting Integrator to snRNA genes, which readily explains the defect in snRNA 3′ end maturation observed after RPAP2 knockdown (Figure 5A). Knockdown of RPAP2 also reduces transcription of *U1 snRNA* and *U2 snRNA* genes to less than 50% (Figure 5D), while transcription of the control Pol III-dependent *5S rRNA* gene remains unaffected. This suggests that the drop in steady-state RNA levels when RPAP2 is knocked down reflects reduced transcription. Accordingly, Pol II occupancy on the U2 snRNA transcription unit is significantly reduced after RPAP2 knockdown (Figure 5E). The effect on transcription may in part be due to the lack of recruitment of Integrator subunits. However, Int4 knockdown rather increases transcription, measured by nuclear run-on and Pol II occupancy (Figure S5D), indicating that the transcription defect is due to the loss of Integrator subunits other than Int4 and Int11 or the loss of RPAP2.

Taken together, these results demonstrate that RPAP2 is required for efficient transcription of snRNA genes by Pol II. Notably, the effect of RPAP2 knockdown on expression of snRNA genes is similar to that caused by mutating Ser7 of the CTD to alanine (Egloff et al., 2007).

### RPAP2 Is a CTD Phosphatase

Mosley and colleagues have identified the yeast homolog of RPAP2, Rtr1, as a CTD phosphatase (Mosley et al., 2009). Since human RPAP2 binds the CTD and affects expression of Pol II-transcribed snRNA genes, we sought to determine whether RPAP2 could also function as a phosphatase. We first analyzed the effect of RPAP2 depletion on CTD phosphorylation by measuring Ser2P, Ser5P, and Ser7P in whole-cell extract after RPAP2 knockdown (Figure 6A). Depletion of RPAP2 using siRNA results in a significant increase in the level of Ser5P but does not affect Ser2P and Ser7P. This result demonstrates that RPAP2 regulates the global level of Ser5P in the cell. Next, we asked whether the Ser5P level was affected on actively transcribing Pol II after RPAP2 knockdown. Since Pol II levels are reduced by RPAP2 knockdown (Figure 5E), the level of Ser5P was related to the level of Pol II (Ser5P/Pol II). After knockdown of RPAP2, the level of Ser5P drastically increases on U2 snRNA genes throughout the whole transcribed region (Figure 6B). This result suggests that RPAP2 dephosphorylates phospho-Ser5 on transcribing Pol II. In order to determine whether RPAP2 acts as a CTD phosphatase in vitro, we performed a phosphatase assay using recombinant RPAP2 purified from bacteria and GST-CTD that has been phosphorylated in vitro by P-TEFb (see the Experimental Procedures). As previously shown (Glover-Cutter et al., 2009), P-TEFb exhibits robust CTD phosphorylation activity in vitro and phosphorylates Ser2, Ser5, and Ser7 (Figure 6C and Figure S6). Addition of increasing amounts of recombinant RPAP2 results in the selective removal of Ser5 phosphate, without affecting Ser2 and Ser7 phosphorylation. As a control, the phosphatase assay was also carried out in the presence of a recombinant RPAP2 unable to bind Pol II. As shown in Figure 6D, the N terminus of the protein (1–334), which is still able to bind Pol II in vivo (Figure 2A), dephosphorylates Ser5 of the CTD as efficiently as does the WT protein, demonstrating that the phosphatase activity is carried by the N terminus of the protein. The same protein carrying point mutations in the DUF408 domain that abolish its association with Pol II (M1 protein in the N-terminal context) has no effect on CTD phosphorylation, demonstrating the specificity of the phosphatase assay. These results demonstrate that RPAP2 has Ser5-specific phosphatase activity in vitro.

### Recruitment of RPAP2 to Protein-Coding Genes Does Not Require Ser7 of the CTD

**Figure 7:**
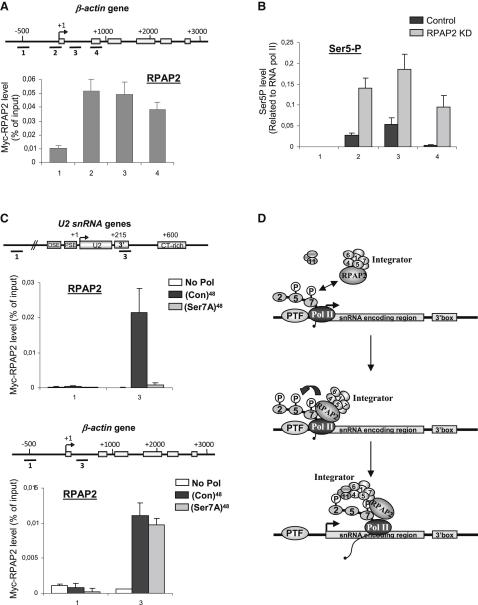
Recruitment of RPAP2 to snRNA Genes through Ser7 Phosphorylation Underpins a Cascade of Events Critical for Proper snRNA Gene Expression (A) qPCR quantitation of ChIP analysis of the *β-actin* gene using antibodies to Myc-RPAP2. Primers used are noted on the diagram of the *β-actin* gene above. (B) qPCR quantitation of ChIP analysis of the *β-actin* gene after RPAP2 knockdown using an antibody against Ser5P, relative to Pol II signal. (C) qPCR quantitation of ChIP analysis of the *β-actin* and *U2 snRNA* genes using antibodies to Myc-RPAP2 after complementation with either [(Con)^48^] and [(S7A)^48^] as indicated. (D) The Pol II CTD is first phosphorylated on Ser5 and then on Ser7 by CDK7. RPAP2 associates with the Pol II CTD after Ser7 phosphorylation and tethers a subcomplex of Integrator to snRNA genes. RPAP2 dephosphorylates Ser5P of the CTD, facilitating transcription and the subsequent recruitment of the Int11 catalytic subunit of Integrator. Error bars indicate the standard deviation obtained from at least three independent experiments.

As has been shown in yeast (Mosley et al., 2009), we find RPAP2 also associated with protein-coding genes, including the *β-actin* gene (Figure 7A). In addition, knockdown of RPAP2 causes a reduction in Pol II levels and a marked increase in the relative level of Ser5 phosphorylation (Figure 7B). These results beg the question, why does mutation of Ser7 of the Pol II CTD specifically affect expression of snRNA genes (Egloff et al., 2007)? ChIP of Myc-RPAP2 on the *U2 snRNA* and *β-actin* genes after complementation of α-amanitin-treated cells with either (Con)^48^ or (S7A)^48^ Pol II indicates that recruitment of RPAP2 to the *U2 snRNA* gene is drastically affected by mutation of Ser7 (Figure 7C). In contrast, RPAP2 is still recruited to the *β-actin* gene when Ser7 is mutated to alanine (Figure 7C). Thus, it appears that recruitment of RPAP2 to protein-coding genes can occur by a mechanism additional or alternative to interaction with phospho-Ser7 of the Pol II CTD.

## Discussion

The CTD of the largest subunit of Pol II serves as a structural binding platform for nuclear factors which, in return, positively or negatively influence the different steps of Pol II transcription and transcription-coupled RNA processing (Buratowski, 2009). The timely recruitment of transcription/RNA processing factors during the transcription cycle is tied to dynamic phosphorylation and dephosphorylation of CTD repeats. Among the three main cotranscriptional phosphorylation events (Ser2/Ser5/Ser7), Ser7P is by far the most obscure. While Ser5P and Ser2P have been described to play key roles in promoter clearance/capping and splicing/elongation, respectively, Ser7P has no clear function in expression of protein-coding genes. So far, the only role for this modification is in expression of the subset of Pol II-transcribed genes encoding snRNAs (Egloff et al., 2007). In this study, we have shown that RPAP2 interacts with the CTD phosphorylated at Ser7 alone, in vitro and in vivo. RPAP2 belongs to the RPAP family, a set of proteins occupying key positions in the protein interaction network of the human Pol II machinery (Jeronimo et al., 2007). Our results indicate that RPAP2 specifically and directly interacts with the Pol II CTD phosphorylated at Ser7.

Importantly, siRNA-mediated knockdown of RPAP2 drastically affects expression of snRNA genes, reducing the transcription rate by Pol II and the efficiency of the 3′ end processing step. These defects are similar to the ones observed after mutation of Ser7 of the CTD (Egloff et al., 2007), suggesting that the loss of RPAP2 may account for the defects observed when Ser7 is mutated. Accordingly, depletion of RPAP2 also causes a consequent loss of the Integrator complex from snRNA genes, as previously shown for Ser7 mutation (Egloff et al., 2007). We confirmed the previously described interaction between RPAP2 and the Int4 and Int6 subunits of the Integrator complex (Jeronimo et al., 2007; Malovannaya et al., 2010). Unexpectedly, the Int11 subunit, thought to carry the endonuclease activity responsible for the cleavage of the 3′ end of pre-snRNAs (Baillat et al., 2005), does not stably associate with RPAP2. Importantly, Int11 binding requires Ser2P, which occurs later in the transcription cycle (Egloff et al., 2009), in addition to Ser7P to efficiently interact with Pol II (Egloff et al., 2010), whereas Int4 requires only Ser7P (Figures S4A and S4B). Together, our data suggest that a subcomplex of Integrator, containing at least Int1, Int4, Int5, Int6, and Int7, and possibly Int3, since Jeronimo et al. identified an interaction between Int3 and RPAP2 (Jeronimo et al., 2007), is loaded onto snRNA genes through RPAP2 and that the catalytic subunit is stably recruited later in the transcription cycle (Figure 7D). This possibility is supported by the finding that Int11 is at its highest level nearest to the 3′ box (Figure 5C and Figure S5E).

The finding that Ser7 is required for the recruitment of a Ser5P phosphatase also has important implications for Ser7 function during snRNA gene transcription, since it suggests that Ser7P tags Pol II for Ser5 dephosphorylation. Accordingly, the Ser5P level drops significantly downstream of the peak of Ser7P on snRNA genes (Egloff et al., 2009). In yeast, abrogation of the two Ser5 phosphatase activities, Rtr1 and Ssu72, causes a transcription defect (Gibney et al., 2008; Mosley et al., 2009; Reyes-Reyes and Hampsey, 2007). The defect in transcription of snRNA genes caused by RPAP2 knockdown may therefore be, at least in part, due to the failure to dephosphorylate Ser5. Ser5 hyperphosphorylation induced by the loss of Ser5 phosphatases has been proposed to either reduce the rate of transcription reinitiation by Pol II, or result in persistent association of the capping enzyme (Reyes-Reyes and Hampsey, 2007), which has been reported to repress transcription (Myers et al., 2002). In addition, early Ser5 dephosphorylation might be a prerequisite for further association of elongation/processing factors, as suggested by our previous observation that Ser5P has a negative impact on Int11 recruitment to the CTD (Egloff et al., 2010). Thus, failure to dephosphorylate Ser5 may also contribute to the failure to efficiently 3′ process snRNA gene transcripts.

Based on these results, we propose a model in which recruitment of RPAP2 to snRNA genes through CTD Ser7 phosphorylation underpins a cascade of events critical for proper gene expression (Figure 7D). Recruitment of RPAP2 results both in stable association of Integrator with snRNA genes and removal of Ser5P, generating the characteristic pattern of CTD phosphorylation found on actively transcribed snRNA genes.

Interestingly, this cascade appears to be specific for snRNA genes, as RPAP2 recruitment to a protein-coding gene is not affected by mutation of Ser7. RPAP2 recruitment to protein-coding genes must therefore involve either additional or entirely different interactions, perhaps reflecting the increased complexity of the transcription apparatus involved in expression of these generally more complex genes.

Finally, we identified the DUF408 domain of RPAP2 as a CTD-interacting domain (CID), raising the possibility that other DUF408-containing proteins may also recognize Ser7P of the Pol II CTD. So far, the DUF408 domain has only been reported to support the phosphatase activity of the yeast Rtr1 protein (Mosley et al., 2009). Our results raise the possibility that the lack of phosphatase activity of the resulting protein could be due to its inability to properly associate with its substrate. Additional studies will therefore be required to precisely map the phosphatase domain and the CID of the human RPAP2.

## Experimental Procedures

### Expression Constructs

To generate pFLAG-RPAP2, RPAP2 cDNA was inserted between HindIII and BamHI sites of pFLAG-CMV-2 (Sigma). pFLAG(1–334), pFLAG(ΔDUF), pFLAG(M1), and pFLAG(M2) expression plasmids were generated by PCR-mediated mutagenesis using pFLAG-RPAP2 as template. To make Myc-RPAP2, three tandem Myc epitopes were cloned at the C terminus of the RPAP2**-**coding region in pCDNA. The level of F-RPAP2 and Myc-RPAP2 relative to endogenous RPAP2 was determined by western blot analysis (Figure S3B). To make GST-RPAP2, the coding region of RPAP2 was cloned into pGEX-6P-1. Six histidines were added to the 3′ end of the GST-RPAP2 by PCR. The identity of all plasmid constructs was verified by sequence analysis.

### Immunoprecipitation

HeLa cells were washed in TBS buffer (150 mM NaCl, 40 mM Tris-HCl [pH 7.5]), resuspended in 500 ml of NET-2 buffer (200 mM NaCl, 0.1% NP-40, 50 mM Tris-HCl [pH 7.5]), and sonicated. Cell debris was removed by ultracentrifugation, and supernatant was incubated with anti-Flag M2 affinity gel (Sigma). After immunoprecipitation, beads were washed six times with NET-2 buffer. Associated proteins were eluted using Flag Peptide (Sigma) and analyzed by SDS-PAGE or silver staining.

### Chromatin Immunoprecipitation

HeLa or 293 cells were transfected using Lipofectamine (Invitrogen) before being subjected to ChIP analysis (Medlin et al., 2005). ChIP samples were analyzed by qPCR using QuantiTect SYBR Green PCR (QIAGEN). Regions analyzed are described in the Supplemental Experimental Procedures. Error bars indicate the standard deviation from at least three independent experiments. Difference in ChIP profile between the profiles obtained using either the Flag or Myc epitopes may be due to differences in detection or cell lines.

### Nuclear Run-On Analysis

Nuclear run-on analysis was carried out as previously described (Medlin et al., 2003). For analysis of endogenous *U2 snRNA* genes, two contiguous 80-mer oligos complementary to the region 208–367 of the *U2 snRNA* gene sequence (Accession number U57614) were used, taking the first base pair of the *U2 snRNA* coding region as 1. For analysis of the *U1 snRNA* genes, two 80-mer oligos complementary to the region 5–164 of the *U1 snRNA* coding region were used, and for *5S RNA* gene, one 80-mer oligo complementary to the region 32–112 of the 5S RNA was used. U1prom, which is complementary to nucleotides −92 to −13 of the *U1 snRNA* gene sequence, and U2prom, which is complementary to nucleotides −268 to −189, were used as negative controls. Hybridization signals were quantified by phosphorimager, corrected with background level, and related to the level of 5S RNA.

### siRNA-Mediated Knockdown

siRNAs targeting RPAP2 were purchased from Dharmacon and transfected using Lipofectamine 2000 (Invitrogen) according to the manufacturer's instructions.

### Western Blot Analysis

Western blotting was carried out as described previously (Medlin et al., 2003) using antibodies against Rpb1 (H-224, Santa Cruz), CTD Ser2P, Ser5P, Ser7P (Chapman et al., 2007), Int4 (Bethyl laboratories), Int11 (Dominski et al., 2005), Flag (M2, Sigma), RPAP2 (17401-1-AP, Proteintech), Myc epitope (sc-764X, Santa Cruz), and actin (sc-1615, Santa Cruz).

### CTD Complementation Assay

293 cells were transfected with α-amanitin-resistant Rpb1 constructs (WT, Con^48^, or Ser7A^48^), and endogenous Rpb1 was turned over by the addition of α-amanitin (Medlin et al., 2003). ChIP was carried out 48 hr after the addition of α-amanitin.

### RT-PCR Analysis

Quantitative RT-PCR was performed on total RNA isolated from WT or RPAP2-depleted HeLa cells. Total RNA (2 μg) was reverse transcribed with random hexamers. U2 pre-snRNA and RPAP2 mRNA were quantitated by real-time PCR analysis using specific primers (primers amplify the region +61/+206 of the U2 gene [accession number U57614], and +91/+224 of the RPAP2 gene [accession number NM_024813]). The results were corrected to the level of 7SK snRNA (primers amplify +11/+190 [accession number NR_001445]). Error bars indicate the range of values from three independent experiments.

### RNase Protections

RNase protection experiments and the U2 riboprobe used in this study have been described previously (Medlin et al., 2003).

### Glycerol Gradient Fractionation

Eluate from Flag-IP or HeLa cell extract (in 50 mM Tris-HCl 7.5, 200 mM NaCl, 1 mM EDTA, 1 mM DTT, 1 mM PMSF) were loaded onto a 5%–45% glycerol gradient, ultracentrifuged for 14 hr at 41000 rpm in a SW41 rotor, and fractionated into 0.5 ml fractions.

### CTD Binding Analysis

In vitro phosphorylation of the GST-CTDs and GST-CTD pull-downs were performed as described previously (Egloff et al., 2007). For the CTD peptide binding assay, CTD peptides were synthesized (Cambridge Peptides) with two repeats of YSPTSPS or YSPTSPA and amino-terminal biotinylation. Streptavidin-coated Sepharose beads (20 μl) were resuspended in 0.1 M KCl HEGN, mixed with 10 μg of biotinylated peptides, and incubated at 4°C for 1 hr. After washing four times with 0.1 M KCl HEGN, 1 μg of recombinant RPAP2 protein was added and the mixture was incubated at 4°C for 3 hr. After washing the beads five times with 1 ml of 0.3 M KCl HEGN, protein-bound beads were boiled and loaded directly on SDS-PAGE gels.

### Phosphatase Assay

Endogenous P-TEFb was purified from G3H cells stably expressing HA-CyclinT1 (Nguyen et al., 2001) and was subsequently used for kinase reactions. Briefly, GST-CTD (Egloff et al., 2007) was incubated with purified P-TEFb for 1 hr at 30°C in kinase buffer (150 mM NaCl, 40 mM HEPES [pH 7.6], 10 mM MgCl_2_, 5 mM DTT, and 500 μM ATP). Kinase reactions were stopped by removal of the unincorporated ATP through a Bio-Spin 6 Tris column (Biorad). Phosphorylated CTD was then incubated with recombinant RPAP2 protein in phosphatase buffer (50 mM Tris-HCl [pH 6.5], 10 mM MgCl_2_, 20mM KCl, and 5 mM dithiothreitol) for 1 hr. Reactions were stopped by the addition of SDS loading buffer.

## Supplemental Information

Document S1. Figure S1, Figure S2, Figure S3, Figure S4, Figure S5, Figure S6, Table S1, Table S2, Supplemental Experimental Procedures, and Supplemental References
