# Supplementary material for: Ser7 Phosphorylation of the CTD Recruits the RPAP2 Ser5 Phosphatase to snRNA Genes
Source: Mol Cell. 2012 Jan 13;45(1):111–22. doi: 10.1016/j.molcel.2011.11.006 (PMC3262128; doi:10.1016/j.molcel.2011.11.006)
Supplement: Document S1. Figure S1, Figure S2, Figure S3, Figure S4, Figure S5, Figure S6, Table S1, Table S2, Supplemental Experimental Procedures, and Supplemental References [file mmc1.pdf]

Molecular Cell, Volume *45*

Supplemental Information

Ser7 Phosphorylation of the CTD Recruits the RPAP2 Ser5 Phosphatase to snRNA Genes

Sylvain Egloff, Justyna Zaborowska, Clélia Laitem, Tamás Kiss, and Shona Murphy

Inventory of Supplemental Information (supplemental information contains Figure S1 to S6, Table S1 and S2, Supplemental experimental procedures and supplemental references).

<u>Figure S1, related to Figure 1</u>
This figure describes the data from the proteomic analysis of the two distinct complexes identified in Figure 1C.
Figure S1A shows a silver stain of the proteins in the two complexes that were subjected to mass spectrometry.
Figure S1B,C integrates the data from Mass Spectrometry with previous data on proteins interacting with Pol II and RPAP2.

<u>Figure S2, related to Figure 2</u>
This figure shows the cellular localisation of the ectopically-expressed proteins described in Figure 2.

<u>Figure S3, related to Figure 3</u>
Panel (A) of this figure shows the association of Myc-tagged RPAP2 with the *U2 snRNA* genes, as measured by ChIP, in comparison to the association of Flag-tagged RPAP2 shown in Figure 3B.
Panel (B) of this figure shows that the ectopically-expressed, epitope-tagged versions of RPAP2 are expressed at approximately the same level as the endogenous protein.

<u>Figure S4, related to Figure 4</u>
This Figure shows that Int4 binds to the Pol II CTD only when the CTD is phosphorylated and Ser7 is not mutated, in common with RPAP2, as shown in Figure 4.

<u>Figure S5, related to Figure 5</u>
Panel (A) of this figure shows the effect of knockdown of RPAP2 on the level of Int5 and Int11, shown in Figure 5C, relative to the effect on Pol II, show in Figure 5E. This supports the notion that loss of Int5 and Int11 is not solely due to loss of Pol II.
Panels (B), (C), (D) and (E) show the effect of knock down of Int4 on processing of transcripts of *U2 snRNA* genes, transcription, Pol II levels and Int11 association, as a comparison to the effects of RPAP2 knockdown described in Figure 5A-E.

<u>Figure S6, related to Figure 6</u>
This figures shows that antibodies to phospho-Ser2, -Ser5 and -Ser7 recognise the CTD specifically after in vitro phosphorylation with purified P-TEFb. This in vitro-phosphorylated CTD was used for the experiments in Figure 6C,D.

<u>Table S1 and Table S2 (related to Figure 1)</u> give the proteins in the two complexes identified by Mass Spectrometry.

A

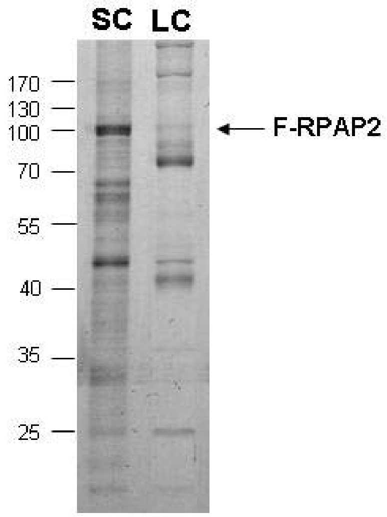

Figure S1 (related to Figure 1). Identification of RPAP2-interacting proteins in the SC and the LC. (A) Silver staining of RPAP2-containing small (SC) and large (LC) complexes. Proteins from the SC and the LC were identified by mass spectrometry (See Table S1 and S2).

B

Protein name
POLR2A
POLR2B
POLR2E
POLR2G
POLR2H
XAB1/GPN1
GPN3
PPP2R1A
DDX3X

C

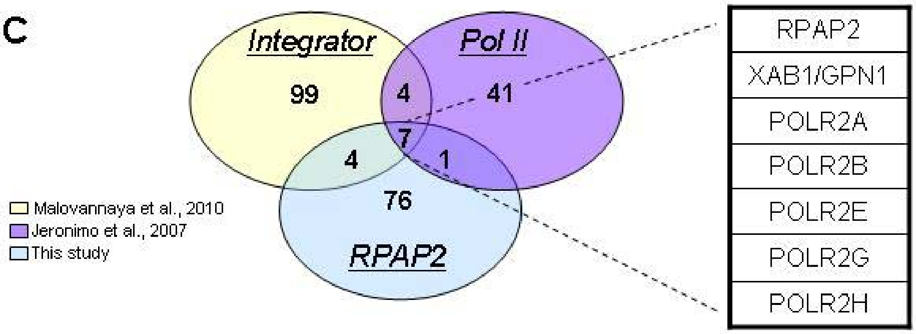

Figure S1. (B) List of Integrator-interacting proteins (Malovannaya et al., 2010) found to interact with RPAP2 (from Table S1). **(C)** Diagram showing the relationships among Integrator, Pol II and RPAP2-associated proteins. The list of proteins interacting with Pol II, Integrator and RPAP2 is indicated on the right.

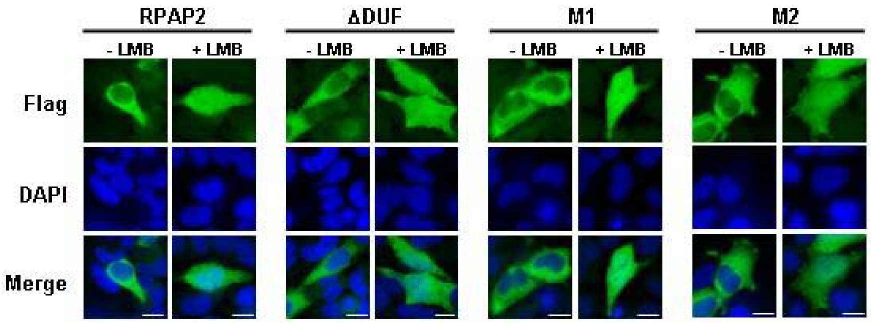

Figure S2 (related to Figure 2). F-RPAP2 proteins shuttle between nucleus and cytoplasm in a CRM1-dependent manner. Localization of F-RPAP2 proteins with or without treatment with leptomycin B (LMB, 20ng/ml) for 2 hours was determined by fluorescence microscopy. Cells were stained with anti-Flag (in green) and DAPI (in blue) to visualize the nuclei. Scale: 10μm.

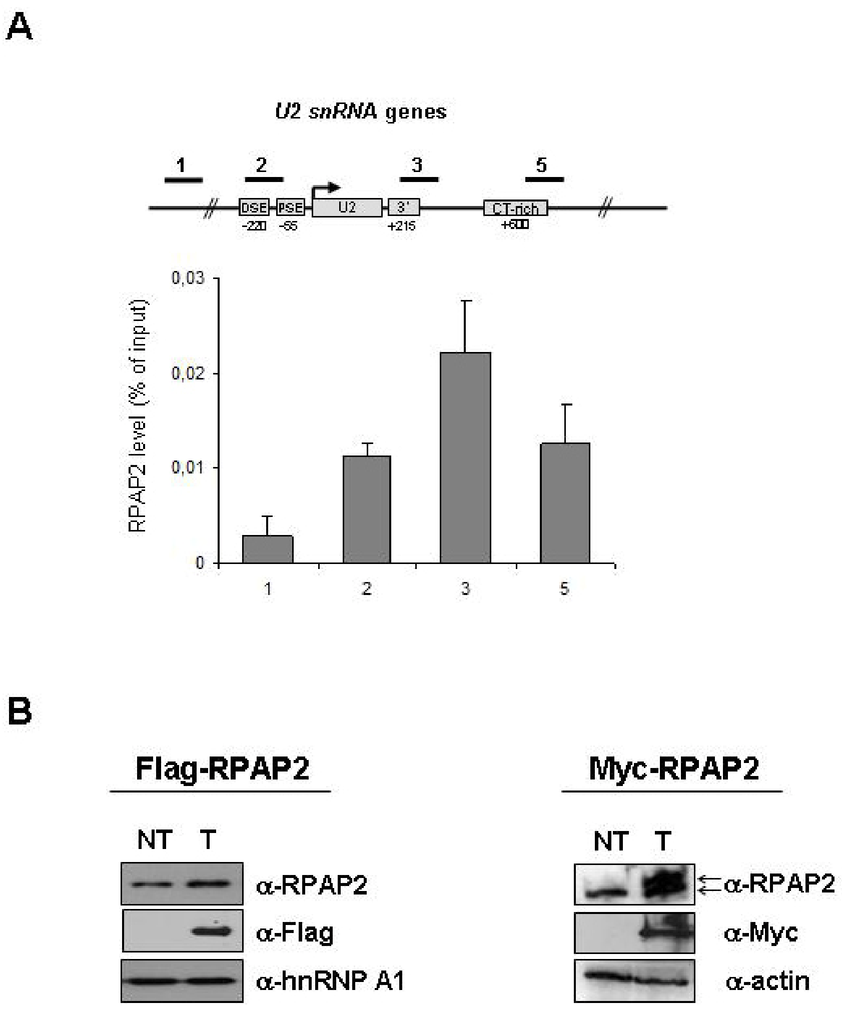

Figure S3 (related to Figure 3). Myc-RPAP2 is recruited to *U2 snRNA* genes and Flag- and Myc-RPAP2 are expressed at the same level as the endogenous protein. (A) ChIP assay was performed in 293 cells using anti-Myc antibody against Myc-RPAP2. The regions amplified are noted on the diagram of *U2 snRNA* genes above. Error bars indicate the standard deviation obtained from at least three independent experiments. **(B)** Extracts from non-transfected (NT) or transfected (T) cells were analyzed by western blot using antibodies directed against RPAP2, Flag, Myc, actin and hnRNP A1 proteins

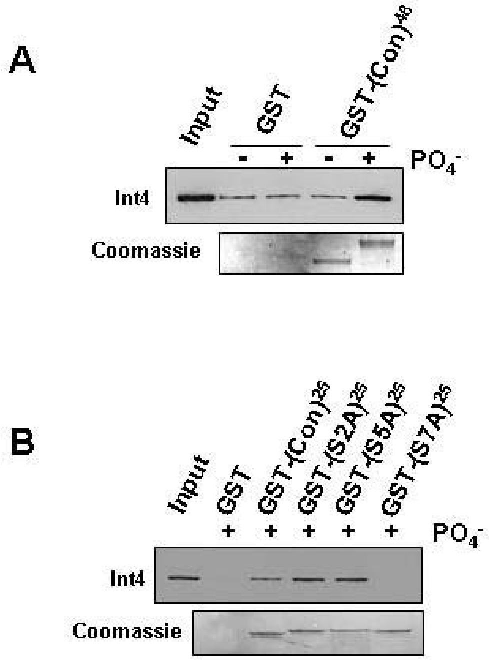

Figure S4 (related to Figure 4). Ser7P is required for Int4 binding to the Pol II CTD. (A) P11 fraction containing the Integrator complex (Egloff et al.; 2007) was incubated with GST alone or GST-CTD $(Con)^{48}$, phosphorylated or not, and Int4 binding was monitored by western blot analysis. The bottom panel represents Coomassie blue staining of the input GST-CTD. **(B)** The P11 fraction containing the Integrator complex was incubated with *in vitro*-phosphorylated GST-CTD and Int4 binding to the WT CTD $(Con)^{25}$ and mutant $(Ser2A/Ser5A/Ser7A)^{25}$ CTDs assayed by Western blot analysis. The bottom panel represents Coomassie blue staining of the input GST-CTD.

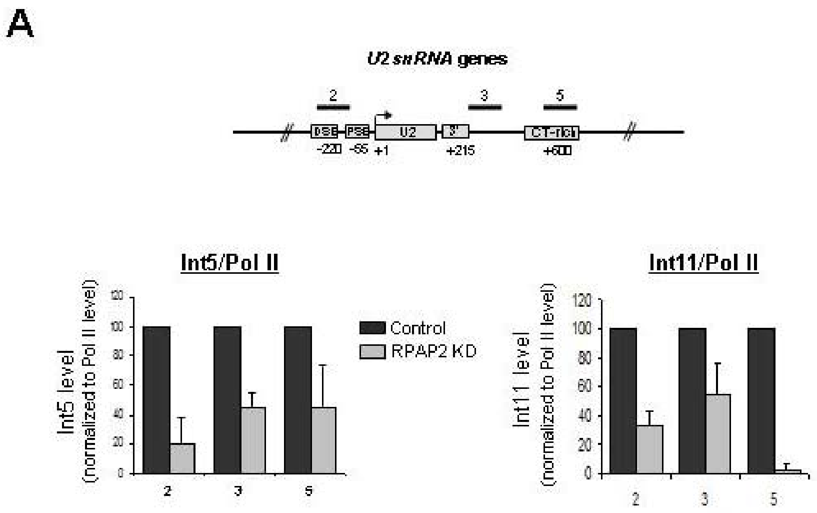

Figure S5 (related to Figure 5). RPAP2 or Int4 knockdown affects Integrator recruitment to and expression of *U2 snRNA* genes. (A) Effect of RPAP2 KD on the Int5 and In11 subunits of Integrator recruitment onto *U2 snRNA* encoding region, relative to Pol II. The regions analyzed are noted on the diagram of *U2 snRNA* genes above. Error bars indicate the standard deviation obtained from at least three independent experiments.

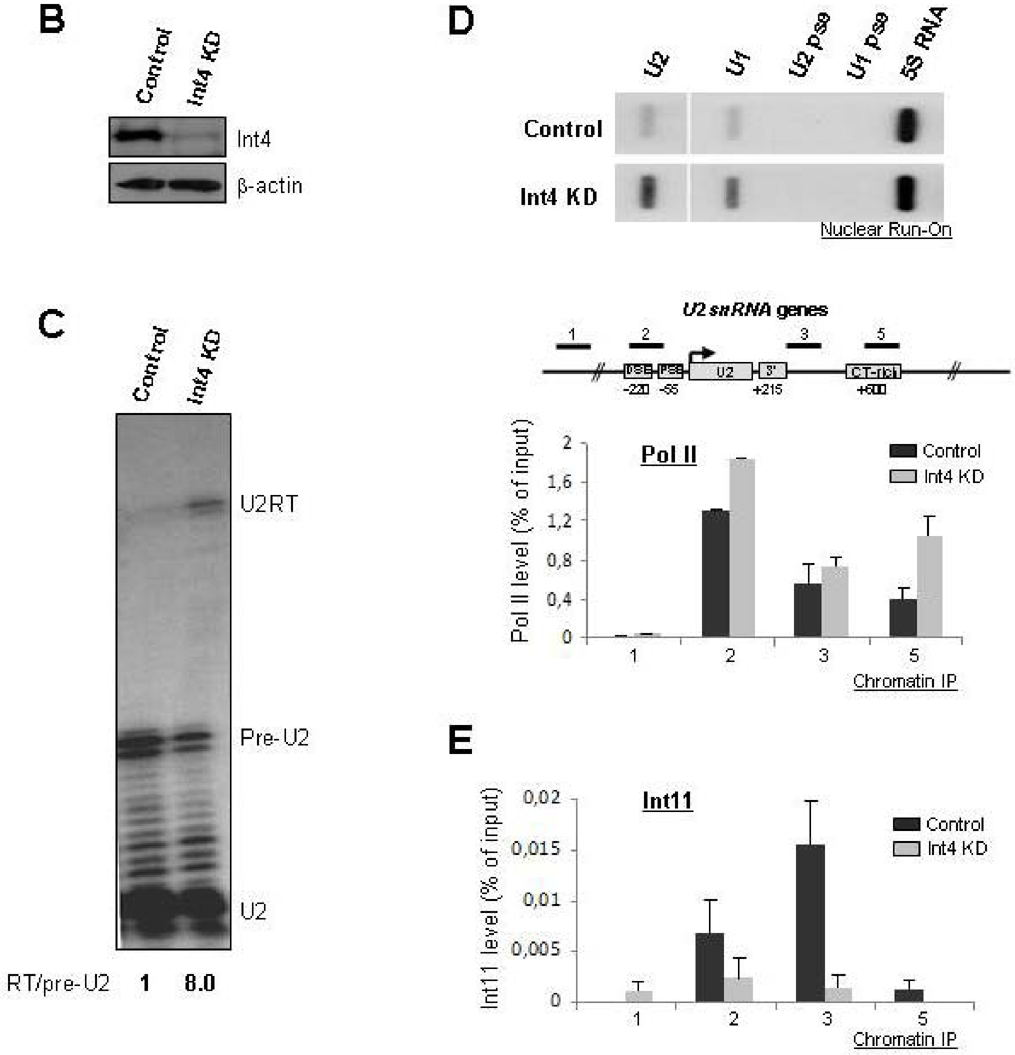

Figure S5. (B) Efficiency of Int4 knockdown was monitored by Western blot using antibodies against Int4 and β-actin. **(C)** RNAse protection analysis of transcripts from endogenous *U2 snRNA* genes in control cells or cells transfected with an siRNA specific for Int4. The different RNA species are noted on the right side. **(D)** (upper panel) Nuclear run-on analysis with and without RNAi-mediated knockdown of Int4 is shown. An oligonucleotide complementary to transcripts from the Pol III-transcribed *5S rRNA* gene was used as a control for the level of transcription. (Bottom panel) qPCR quantitation of ChIP analysis of *U2 snRNA* genes using an antibody against Pol II, after Int4 KD. The regions amplified are shown on the diagram at the top. **(E)** qPCR quantitation of ChIP analysis using an antibody against Int11, after Int4 KD. The regions amplified are the same as in (C). Error bars indicate the standard deviation obtained from at least three independent experiments.

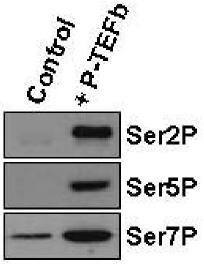

Figure S6 (related to Figure 6). Purified P-TEFb phosphorylates Ser2, Ser5 and Ser7 *in vitro*. Endogenous P-TEFb purified from G3H cells expressing HA-CyclinT1 was used for kinase reaction (see Materials & Methods). The same procedure was performed with HeLa cells as a negative control. Phosphorylation of GST-CTD was visualized by western blot using antibodies against Ser2P, Ser5P and Ser7P.

Table S1. List of the interaction partners of RPAP2 within the SC and the LC
Mascot score, number of peptides, % coverage and emPAI values are indicated for each protein (positive identification: at least 2 unique peptides with length greater than 8 amino-acids, the limit of Mascot score≥60 is indicated).

Small Complex					
	Protein name	Mascot Score	Peptides	% coverage	emPAI
IPI00027251	STK38	444	28	53	2,29
IPI00293375	RPAP2	402	39	49	1,17
IPI00375731	RBM10	367	25	28	0,53
IPI00021439	ACTB	315	13	38	1,16
IPI00470580	GPN3	254	7	28	0,71
IPI00237011	STK38L	243	24	54	1,22
IPI00004511	PFKFB3	222	12	28	0,61
IPI00479743	POTEE	217	9	9	0,19
IPI00413755	TAF4	207	14	19	0,26
IPI00027035	GPN1	206	7	21	0,51
IPI00792677	TUBA1B	184	7	26	0,59
IPI00216049	HNRNPK	181	12	41	0,64
IPI00003865	HSPA8	176	12	25	0,66
IPI00012079	EIF4B	173	21	33	0,51
IPI00215965	HNRNPA1	167	12	30	0,59
IPI00013881	HNRNPH1	156	8	23	0,34
IPI00003362	HSPA5	141	10	20	0,35
IPI00291802	LMO7	128	5	8	0,13
IPI00550655	SPIN1	127	9	41	0,82
IPI00292000	PRPF31	127	11	23	0,48
IPI00027834	HNRNPL	125	5	12	0,18
IPI00104050	THRAP3	124	19	15	0,22
IPI00106955	C11orf84	117	5	20	0,42
IPI00013415	RPS7	109	3	14	0,38
IPI00441473	PRMT5	107	10	19	0,28
IPI00179964	PTBP1	106	7	22	0,37
IPI00107745	LUC7L3	104	4	14	0,23
IPI00007765	HSPA9	98	8	19	0,16
IPI00301503	TRA2B	97	4	18	0,24
IPI00008433	RPS5	95	4	14	0,36
IPI00419373	HNRNPA3	94	5	25	0,31
IPI00012202	WDR77	93	5	9	0,33
IPI00418471	VIM	90	4	10	0,22
IPI00011253	RPS3	86	7	38	0,49
IPI00299155	PSMA4	84	2	17	0,27
IPI00215637	DDX3X	84	4	8	0,16
IPI00396485	EEF1A1	79	8	21	0,24
IPI00218895	POLR2G	78	3	22	0,44
IPI00290566	TCP1	77	3	8	0,13
IPI00015922	DDX26	77	3	5	0,12
IPI00553169	FLNA	74	4	2	0,06
IPI00738381	EEF1G	71	2	5	0,15
IPI00396435	DHX15	70	4	6	0,13
IPI00328840	THOC4	69	3	20	0,29
IPI00301936	ELAVL1	68	4	12	0,32

IPI00019459	TAB1	66	3	11	0,14
IPI00026612	PPM1B	66	3	9	0,07
IPI00007334	ACIN1	64	3	3	0,05
IPI00413672	BCLAF1	63	13	17	0,11
IPI00009328	EIF4A3	63	6	26	0,36
IPI00063234	PRKAR2A	61	3	11	0,09
IPI00027626	CCT6A	60	3	9	0,13
IPI00005613	U2AF1	60	2	5	0,28
IPI00018140	SYNCRIP	57	5	13	0,17
IPI00444262	NCL	57	2	5	0,12
IPI00396218	SCYL2	53	7	6	0,07
IPI00010720	CCT5	53	2	5	0,06
IPI00964686	HNRNPAB	52	4	21	0,12
IPI00386043	C1orf27	52	2	7	0,07
IPI00554737	PPP2R1A	51	4	10	0,12
IPI00297779	CCT2	48	2	6	0,06
IPI00396378	HNRNPA2B1	47	8	34	0,46
IPI00939530	SRSF7	46	5	48	0,24
IPI00302927	CCT4	45	2	5	0,04
IPI00216592	HNRNPC	33	3	16	0,12
IPI00010204	SRSF3	31	4	26	0,43
IPI00030243	PSME3	30	3	15	0,27
IPI00514032	CCT3	26	3	21	0,12

Large Complex					
	**Protein name**	**Mascot Score**	**Peptides**	**% coverage**	**emPAI**
IPI00218895	POLR2G	123	6	44	1,06
IPI00003309	POLR2H	121	4	39	0,5
IPI00021439	ACTB	106	11	41	0,67
IPI00293375	RPAP2	99	10	29	0,37
IPI00479743	POTEE	68	5	5	0,06
IPI00291093	POLR2E	45	2	14	0,15
IPI00022774	VCP	44	2	4	0,04
IPI00021428	ACTA1	36	8	23	0,29
IPI00031627	POLR2A	33	5	3	0,03
IPI00003865	HSPA8	30	2	8	0,05
IPI00014424	EEF1A2	28	2	4	0,07
IPI00216518	IL7R	27	2	4	0,12
IPI00007188	SLC25A5	24	2	8	0,11
IPI00025447	EEF1A1	23	3	7	0,16
IPI00027808	POLR2B	15	3	3	0,06

Figure S1, Table 2. List of the interaction partners of RPAP2 within the SC and the LC after removal of frequent Flag-IP contaminant proteins (*Dunham et al., 2011*)**.** Mascot score, number of peptides, % coverage and emPAI values are indicated for each protein (positive identification: at least 2 unique peptides with length greater than 8 amino-acids, Mascot score≥60).

Small Complex					
	**Protein name**	**Mascot Score**	**Peptides**	**% coverage**	**emPAI**
IPI00293375	RPAP2	402	39	49	1,17
IPI00470580	GPN3	254	7	28	0,71
IPI00413755	TAF4	207	14	19	0,26
IPI00027035	GPN1	206	7	21	0,51
IPI00216049	HNRNPK	181	12	41	0,64
IPI00291802	LMO7	128	5	8	0,1
IPI00292000	PRPF31	127	11	23	0,48
IPI00013415	RPS7	109	3	14	0,38
IPI00179964	PTBP1	106	7	22	0,37
IPI00107745	LUC7L3	104	4	14	0,23
IPI00301503	TRA2B	97	4	18	0,24
IPI00215637	DDX3X	84	4	8	0,16
IPI00218895	POLR2G	78	3	22	0,44
IPI00290566	TCP1	77	3	8	0,13
IPI00015922	DDX26	77	3	5	0,12
IPI00328840	THOC4	69	3	20	0,29
IPI00301936	ELAVL1	68	4	12	0,32
IPI00019459	TAB1	66	3	11	0,14
IPI00026612	PPM1B	66	3	9	0,07
IPI00413672	BCLAF1	63	13	17	0,11
IPI00009328	EIF4A3	63	6	26	0,36
IPI00063234	PRKAR2A	61	3	11	0,09
IPI00027626	CCT6A	60	3	9	0,13

Large Complex					
	**Protein name**	**Mascot Score**	**Peptides**	**% coverage**	**emPAI**
IPI00218895	POLR2G	123	6	44	1,06
IPI00003309	POLR2H	121	4	39	0,5
IPI00293375	RPAP2	99	10	29	0,37

SUPPLEMENTAL EXPERIMENTAL PROCEDURES

Primers used for chromatin immunoprecipitation analysis

Regions amplified by each pair of primers on the *RNU2* locus (Accession Number U57614) and the *β-actin* gene (Accession number M10277) are indicated relative to the transcription start site.

	U2 snRNA gene
1	-2000/-1819
2	-40/+35
3	+259/+355
4	+480/+588
5	+647/+753
6	+765/+906
7	+1432/+1573

	β-actin gene
1	-609/-479
2	-169/-10
3	+222/+361
4	+742/+911

Immunofluorescence

Transfected HeLa cells were fixed in 4% paraformaldehyde in PBS for 30 minutes at room temperature and permeabilized overnight at 4°C in 70% ethanol. Coverslips were then blocked in 1% BSA diluted in PBS for 45 minutes and incubated with the primary antibody (anti-Flag) diluted in the blocking solution, for 2 hours at room temperature. After three washes with PBS, cells were incubated for 1 hour with the secondary antibody (anti-mouse, FITC), diluted in the blocking solution and washed three times with PBS. Coverslips were mounted in Moviol DAPI. Images were captured with a CoolSnap ES camera (Roper Scientific, Tucson, AZ) mounted on a microscope (model DMRA, Leica, Deerfield, IL) with a Leica 100x plan Apo 1.4.

Mass spectrometry analysis

The sample was run just 5mm into the top of a 4-12% gradient gel**.** The top of the gel was excised and chopped into $1mm^3$ cubes and destained with 25 mM ammonium bicarbonate in 50% acetonitrile. The gel pieces were reduced with 10 mM DTT at 37°C for 30 minutes and alkylated with 55mM iodoacetamide for 1 hour and digested with trypsin for 16 hours. Digests were stopped by addition of acetic acid and the peptides desalted on an in-house manufactured C18 device.

Tryptic digests were analysed on a Thermo LTQ orbitrap mass spectrometer coupled to a Dionex U3000 nano HPLC system. Samples were analysed on 120 minutes LC/MS/MS gradient on a 15 cm long by 75 micron inner diameter nano-column packed in-house with C18 material (Reprosil-Pur C18-Aq, 3 micron diameter beads, Dr Maisch).

Mass spectrometer was run in a data-dependent "Top 5" method in which 2+, 3+ and 4+ ions were selected for fragmentation.

Data were analysed using Mascot searched against IPI human (ipi.HUMAN.v3.76). Mass tolerance for precursor was 20 ppm and fragment mass tolerance was 0.5 Da. Fixed

modification was defined as carbamidomethyl (C) and variable modifications allowed were oxidation (M), acetylation (protein N-terminal), Deamidation (NQ) and phospho (STY).
Criteria for acceptance of Mascot identification were defined as followed: Mascot score=60, with at least 2 unique peptides with length of greater than 8 amino-acids (Table S1). Frequent Flag-IP contaminant proteins were removed from our dataset (Table S2), according to the list published by Dunham et al, (2011).

SUPPLEMENTAL REFERENCES

Dunham, W. H., Larsen, B., Tate, S., Badillo, B. G., Goudreault, M., Tehami, Y., Kislinger, T., and Gingras, A. C. (2011) A cost-benefit analysis of multidimensional fractionation of affinity purification-mass spectrometry samples. Proteomics *11*, 2603-2612.
